# Professor Silliman

**Published:** 1849-06

**Authors:** 


### PROFESSOR SILLIMAN.

The newly elected Professor of Chemistry in the University of Louisville gratified his friends in this city with a short visit a few days since. We learn that the Board of Trustees, at his suggestion, have ordered large additions to the already excellent suite of chemical apparatus in the school. The impression made by Professor Silliman upon his new friends here is all that his friends in New England could desire. No one who has had the advantage of his acquaintance doubts that he is a most valuable acquisition to the school and the city.

